# Sexual Dimorphism of Heart Rate Variability in Adolescence: A Case-Control Study on Depression, Anxiety, Stress Levels, Body Composition, and Heart Rate Variability in Adolescents with Impaired Fasting Glucose

**DOI:** 10.3390/ijerph17082688

**Published:** 2020-04-14

**Authors:** Charikleia Stefanaki, Athanasios Michos, George Latsios, Dimitrios Tousoulis, Melpomeni Peppa, Paraskevi Zosi, Dario Boschiero, Flora Bacopoulou

**Affiliations:** 1 Center for Adolescent Medicine and UNESCO Chair on Adolescent Health Care, First Department of Pediatrics, School of Medicine, National and Kapodistrian University of Athens, Aghia Sophia Children’s Hospital, 115 27 Athens, Greece; amichos@med.uoa.gr (A.M.); fbacopoulou@med.uoa.gr (F.B.); 2 Department of Pediatrics, General Hospital of Nikaia “Agios Panteleimon”, 184 54 Piraeus, Greece; zosiv@yahoo.gr; 3 First Cardiology Department, School of Medicine, National and Kapodistrian University of Athens, Hippokration Hospital, 115 28 Athens, Greece; glatsios@gmail.com (G.L.); tousouli@med.uoa.gr (D.T.); 4 Endocrine Unit, Second Department of Internal Medicine Propaedeutic, Research Institute and Diabetes Center, Attikon University Hospital, National and Kapodistrian University of Athens, 115 28 Athens, Greece; moly6592@yahoo.com; 5 BIOTEKNA Biomedical Technologies, 30020 Venice, Italy; dario.boschiero@gmail.com

**Keywords:** adolescence, impaired fasting glucose, body composition, heart rate variability, BIA, DASS21

## Abstract

Prediabetes in the form of impaired fasting glucose, impaired glucose tolerance, or both is considered as a preliminary stage for the onset of diabetes and diabetic complications. Hormonal fluctuations in adolescence are accompanied by body composition modifications, which are associated with insulin resistance and subclinical inflammation. Bioimpedance (BIA) accurately evaluates body composition, and heart rate variability (HRV) assesses cardiac autonomic function, which are frequently afflicted by insulin resistance. We aimed at evaluating the effect of glycemic status on mental stress, anxiety, and depression status in adolescents with impaired fasting glucose, body composition, and HRV parameters. This is a case-control study to evaluate the effect of the hyperglycemia on depression, anxiety, and stress levels (DASS21 questionnaire), body composition (BIA-ACC—BIOTEKNA©), and HRV (PPG Stress Flow—BIOTEKNA©), between euglycemic adolescents (euglycemic group) and adolescents with impaired fasting glucose (prediabetic group), aged 12–20 years. No differences were found between the prediabetic (*n* = 13) and the euglycemic (*n* = 16) groups in the outcome measures, possibly due to the number of participants. Interestingly, females, irrespective of their glycemic status, exhibited altered sympathovagal function as revealed by impaired HRV. In the euglycemic group, HRV parameters were significantly correlated and in line with the DASS21 scores, but in the prediabetic group, similarities to those of adults were observed. Impaired fasting glucose had no impact on mental health, body composition, or HRV parameters in adolescents. HRV parameters were impaired in females, irrespective of their glycemic status. This finding implies that females seem to be more prone to stress disorders, even from a young age. Future studies are needed to confirm these findings.

## 1. Introduction

Prediabetes entails a spectrum of subclinical, proinflammatory processes, which include either isolated impaired fasting glucose (IFG), impaired glucose tolerance (IGT), or both (IFG/IGT). Hyperglycemia is linked to disruption of the glucose homeostasis in the skeletal muscles and the liver. When there is dysfunction in these tissues, they generate subclinical proinflammatory processes, which herald the onset of prediabetes complications with different requirements for prevention and treatment if development to full-blown diabetes occurs [1,2].

During adolescence, along with the advancement of sexual maturation, hormonal fluctuations promote lean mass, fat mass, and bone mass increases in different proportions between the sexes, rendering adolescents prone to insulin resistance. The latter seems to play a pivotal, reciprocal role in the observed changes in body composition throughout adolescence [3].

**Figure 1:**
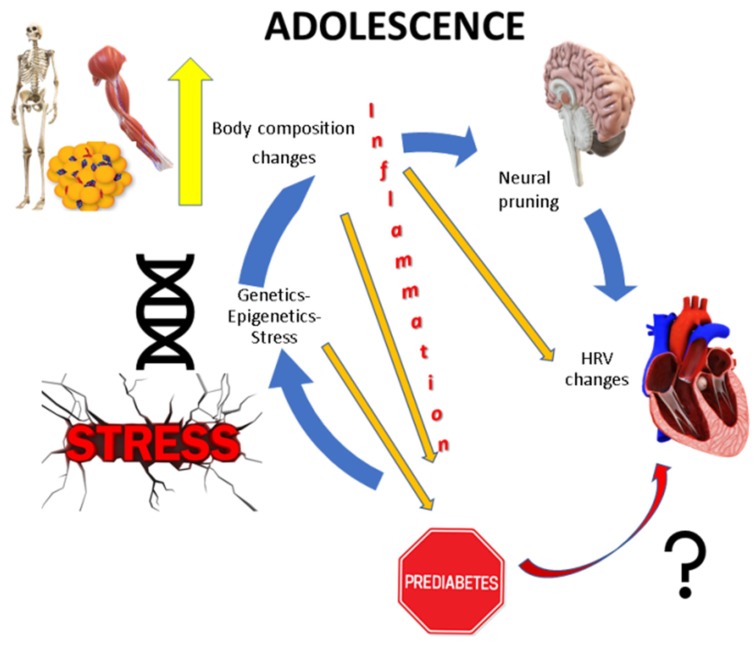
Concept of the study.

Many studies have demonstrated significant correlations between the hyperactivity of hypothalamic–pituitary–adrenal (HPA) axis, weight gain, and subsequent accumulation of fat tissue [4,5,6]. Thus, overactivation of the stress system appears to contribute to the increase of fat tissue, primarily through upregulation of the HPA axis. Vice versa, increase of fat mass per se seems to constitute a chronic proinflammatory, stressful state that also causes HPA axis imbalance. Additionally, the subclinical inflammation of obesity that contributes to the derangement of the metabolic equilibrium suggests that the proinflammatory cytokines secreted by the adipocytes hold a potentially important pathogenetic role [7]. The proinflammatory molecules, either produced by the adipocytes or by physical and mental stress, contribute to body composition disorders, with detrimental implications in adult health [8] (Figure 1).

Prediabetes is the stage when the diabetic complications begin. There is a significant association between cardiovascular events and IGT, whereas this correlation is less clear in cases of IFG [9,10]. Studies investigating cardiovascular events and cardiac autonomic nervous dysfunction due to prediabetes show controversies, particularly in isolated IFG. It has been stated that altered cardiac autonomic function is present in both IGT and diabetes but not in patients with isolated IFG [11]. The most commonly used methods for the diagnosis of cardiac autonomic function is based on heart rate variability (HRV) assessment, which is the physiological oscillation in the time interval between heartbeats and enables the independent measurement of the parasympathetic and sympathetic components of the autonomic nervous system [12]. Sympatho-adrenal medullary system (SAM) and HPA axis reactivity seem to predict future health and disease outcomes. Exaggerated and blunted responses predict different health and disease outcomes. Reactivity-related health and disease outcomes are both physical and mental. Dysregulation of stress reactivity may represent a mechanism by which psychological stress contributes to the development of future health and disease outcomes [6]. Available data on autonomic nervous system dysfunction in prediabetic adolescents are scarce. The majority of the few studies that evaluate body composition and HRV in prediabetic patients involve adults and they report associations between obesity, proinflammatory metabolic parameters and HbA_1c_, and autonomic nervous system dysfunction; however, they have no direct relation to prediabetes [13,14].

The aim of this study was to explore the differences between euglycemic adolescents and prediabetic adolescents with IFG in body composition, HRV parameters, and mental aspects along with depression, anxiety, and stress levels.

## 2. Methods

### 2.1. Study Design—Setting—Procedures

**Figure 2:**
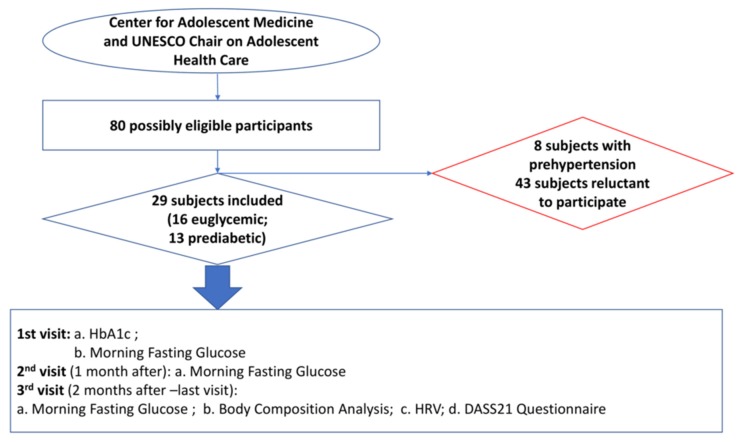
Participants’ flow chart and study procedure.

This case-control study was approved by the National and Kapodistrian University of Athens, Faculty of Medicine Ethics Committee and by the Ethics Committee of the Aghia Sophia Children’s Hospital (repository number: 28931/11.02.2015) and was in accordance with the Helsinki Declaration for human studies [15] and European Medicines Agency Guidelines for Good Clinical Practice. The participants, as well as their legal guardians, gave informed, written consent after explanation of the purpose and the nature of all the procedures used in the study. The privacy rights of human subjects were conserved. The study took place at the Center for Adolescent Medicine (CAM) and United Nations Educational, Scientific and Cultural Organization (UNESCO) Chair on Adolescent Health Care of the First Department of Pediatrics, National and Kapodistrian University of Athens, in Athens, Greece, from March 2015 to January 2016. Study participants were screened for diabetes monthly during each of their three visits. During their last (third) visit, adolescents underwent assessment of their body composition and heart rate variability along with their depression, anxiety, and stress levels, as shown in Figure 2.

### 2.2. Participants

Adolescent males and females, aged 12–20 years, during their routine visits at the CAM, were evaluated for eligibility to enter the study based on inclusion and exclusion criteria.

Adolescents, defined by a breast Tanner stage equal to III or IV in females, and testicular Tanner stage equal to III or IV in males, were included in the study if they were willing to undergo measurements and intervention procedures and were either euglycemic or prediabetic, based on their serum blood tests and according to the American Diabetes Association (ADA) criteria: a. hemoglobin A1c (Hb1_Ac_) ranging from 5.7% to 6.4%; and/or b. fasting serum glucose concentrations ranging from 100 to 125 mg/dL.

Exclusion criteria were: a. previous diagnosis of diabetes (established diagnosis of any other type of diabetes); b. treatment with medication that could elevate or normalize blood glucose such as glucocorticosteroids, antidiabetic drugs, or metformin; c. presence of an established diagnosis of cardiovascular disease (congenital, genetic, or otherwise), hypertension, or prehypertension; d. psychiatric disorder; and e. psychiatric disease requiring consultation and/or pharmacologic treatment. After written consent was obtained, study participants were assessed at baseline and at two sequential monthly visits to the CAM.

Baseline demographic characteristics included: age, biological sex, weight, height, body mass index (BMI) z-score, presence of positive family history for diabetes, i.e., first- or second-degree relatives with diabetes, and single-parent family structure, i.e., divorced, separated, or widowed legal guardians.

### 2.3. Measurements

#### 2.3.1. Diabetes Screening

Blood was drawn from each participant in the morning at 8 a.m., after a 10-h overnight fast. During the visits, adolescents would not be subject to intercurrent illness, recent accident, or extremely stressful events. Blood samples were collected for measurement of serum glucose on each of the three visits (1st, 2nd, 3rd), whereas HbA1c was measured only on the 1st visit.

#### 2.3.2. Body Composition Analysis

Body composition analysis was conducted via bioimpedance (BIA), using the BIA-ACC device (BIOTEKNA©, Biomedical Technologies, Venice, Italy), which has been validated against dual-energy X-ray absorptiometry (DXA) [16,17,18]. Measurements were performed according to the manufacturer’s guidelines, during the 3rd visit, between 8:15 and 8:30 a.m., after an overnight fast, and bladder voiding. Adolescents should not have performed any exercise before measurements. This device applies alternating currents, using two different frequencies, 50 and 1.5 kHz (bi-frequency measurement method), to compute body composition, based on a multicompartment model (2C, 3C, 4C, 5C). Assessment was performed with each participant lying supine on a flat, nonconducting surface, with no contact with metallic elements. Two electrodes were applied on the dorsal surface of the right hand and two electrodes on the dorsal surface of the right foot.

The formulas used for computations have been previously described in detail [18]. For each adolescent, the following body composition parameters were estimated: total body water in Lt and % of total body weight; intracellular body water in Lt and % of total body water; extracellular body water in Lt and % of total body water; fat-free mass in kg, and body weight %; fat mass in kg and body weight %; skeletal muscle mass in kg and fat-free mass %; abdominal adipose tissue in cm^2^; and intramuscular adipose tissue in kg and body weight %. The results were shown in the BioTekna Plus software platform on a local personal computer (PC).

#### 2.3.3. Heart Rate Variability Assessment

Heart rate variability (HRV) was assessed with the use of a photo-plethysmographic (PPG) stress flow device, based on a multi-channel plethysmographic technology applied to the distal ends of the limbs that allows analysis of the overall activity of the autonomic nervous system and of the HRV. The HRV test has a duration of 5 min and allows monitoring of the functionality of the autonomic nervous system and assists in the differential diagnosis of chronic inflammatory and stress-related disorders [19].

Measurements were performed according to the manufacturer’s guidelines during the 3rd visit between 8:00 and 8:15 a.m., for all study participants, to assess average heart rate (mean HR), time domain parameters, such as standard deviation of all normal-to-normal (NN) intervals (SDNN); square root of the mean of the sum of the squares of differences between adjacent NN intervals (RMSSD) and frequency domain parameters, such as power in very low frequency range 0.04 Hz (VLF power) as prefrontal cortex (PFC) activity indicator; power in low frequency range 0.04–0.15 Hz (LF power) as sympathetic activity indicator; power in high frequency range 0.15–0.4 Hz (HF power) as parasympathetic activity indicator; and HRV representation (scatter-heart rate) and bilateral flow bloodstream multi-channel representation {LF (in ms^2^)/HF (ms^2^) ratio}. The results were shown in the BioTekna Plus software platform on a local PC [12,20].

#### 2.3.4. DASS21 (Depression, Anxiety, Stress Scales) Questionnaire

The DASS21 comprises a set of three self-reported scales, designed to measure the perception/physical symptomatology of the negative emotional states of depression, anxiety, and stress, during the past week. It includes 21 items, divided into subscales of seven items each; the depression subscale (items 3, 5, 10, 13, 16, 17, and 21), the anxiety subscale (items 2, 4, 7, 9, 15, 19, and 20), and the stress subscale (items 1, 6, 8, 11, 12, 14, and 18). The questionnaire, validated in Greek [21], was given to all study participants. All responses were ranked on a four-point Likert scale, ranging from 0 (“did not apply to me—never”) to 3 (“applied to me very much or most of the time—almost always”).

### 2.4. Statistical Methods

Statistical significance was set at *p* < 0.05. All data analyses were performed using the SPSS v.21 statistical software (IBM Co., New York, NY, USA). Baseline descriptive characteristics are represented as median values, range, and quartiles for continuous variables and absolute and proportional values (%) for categorical variables. Mann–Whitney U tests were employed for the evaluation of the statistical differences between prediabetic and euglycemic adolescents, as well as between males and females. Pearson’s chi square tests were employed for the frequency group comparisons. Spearman’s rho tests were performed for the evaluation of the variables under study. Cronbach alpha coefficient analyses were performed for every DASS21 subscale, to assess internal consistency.

### 2.5. Sample Size Calculations

We aimed at 12–25 subjects per group as this was a pilot study [22].

## 3. Results

### 3.1. Study Sample

Initially, a total of 80 adolescents, who attended the Center for Adolescent Medicine and UNESCO Chair on Adolescent Health Care over the three-month recruitment period, were deemed eligible to enter the study. Fifty-one adolescents were subsequently excluded and finally 29 adolescents were included in the study; 16 euglycemic (euglycemic group) and 13 prediabetic (prediabetic group) who were found to have IFG for 3 consequent months (Figure 2).

No statistically significant differences in the baseline psychometric and demographic characteristics were found between the two groups, except for the three subsequent measurements of serum glucose concentrations (Table 1). The Cronbach alpha coefficients for DASS21 Subscales internal consistency proved to be extremely reliable (DASS21 Stress: 0.881; DASS21 Anxiety: 0.862; and DASS21 Depression: 0.883). According to the DASS manual, the participants of both groups did not exhibit severe or extremely severe clinical depression, anxiety, or stress [23].

### 3.2. Body Composition Analysis and Heart Rate Variability

No statistically significant differences were found in the body composition parameters or the HRV parameters between the prediabetic and the euglycemic group (Table 2).

**Figure 3:**
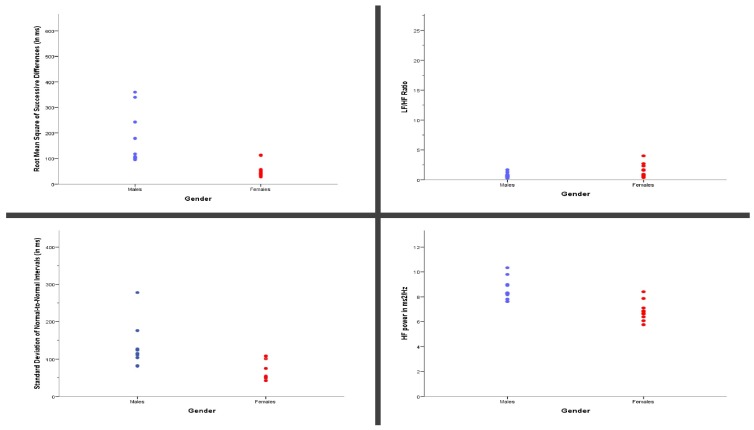
Statistically significant differences in heart rate variability (HRV) parameters between the biological sexes.

However, statistically significant differences were detected in the time and frequency domain parameters of the HRV between males and females, irrespective of the glycemic state. More specifically, the SDNN, RMSSD, and HF power (Figure 3) were decreased in females vs. males ((Median = 75 vs. 113; 48 vs. 106.5; 6.81 vs. 8.27, respectively); Mean Rank = 7.82 vs. 15.83; U = 20; *p* = 0.004/Mean Rank = 8.36 vs. 15.33; U = 26; *p* = 0.013;/Mean Rank = 8.55 vs. 15.17; U = 23; *p* = 0.007, respectively), whereas the LF/HF ratio (Figure 3) was increased in females vs. males ((Median = 1.6 vs. 0.65, respectively); Mean Rank = 15.45 vs. 8.83, U = 28; *p* = 0.019).

No statistically significant differences between males vs. females were found for any other study variables.

### 3.3. Additional Analyses

Analysis of the total sample did not yield any statistically significant correlations; however, when the two groups were analyzed separately, statistically significant correlations were found between several HRV parameters and the scores of DASS21 subscales.

In the euglycemic group, both the DASS21 Depression and Anxiety subscale scores were negatively correlated with LF/HF log (Spearman’s rho = −0.879, *p* = 0.009; Spearman’s rho = −0.781, *p* = 0.038, respectively); LF/HF ratio (Spearman’s rho = −0.879, *p* = 0.009; Spearman’s rho = −0.781, *p* = 0.038, respectively); LF % (Spearman’s rho = −0.919, *p* = 0.003; Spearman’s rho = −0.863, *p* = 0.012, respectively); and were positively correlated with HF % (Spearman’s rho = 0.919, *p* = 0.003; Spearman’s rho = 0.863, *p* = 0.012, respectively).

In the prediabetic group, the DASS21 Stress subscale score was negatively correlated with RMSSD (Spearman’s rho = −0.805, *p* = 0.002); VLF (Spearman’s rho = −0.654, *p* = 0.021); LF % (Spearman’s rho = −0.777, *p* = 0.003); and HF % (Spearman’s rho = 0.787, *p* = 0.002). The DASS21 Anxiety subscale score was negatively correlated with SDNN (Spearman’s rho = −0.585, *p* = 0.046); HF % (Spearman’s rho = −0.609, *p* = 0.036); and positively correlated with LF/HF log (Spearman’s rho = 0.641, *p* = 0.025); LF/HF ratio (Spearman’s rho = 0.602, *p* = 0.038); LF % (Spearman’s rho = 0.63, *p* = 0.028); and HF % (Spearman’s rho = −0.63, *p* = 0.028). In the same group, the DASS21 Depression subscale score was negatively associated with SDNN (Spearman’s rho = −0.585, *p* = 0.046).

## 4. Discussion

In this study, we examined the differences between euglycemic and prediabetic adolescents in their body composition and HRV, with concurrent evaluation of their depression, anxiety, and stress levels. No differences were found between the euglycemic and the prediabetic group, whereas female sex seemed to have an impact on HRV, irrespective of the glycemic status. Additionally, statistically significant associations were revealed between time and frequency domains of the HRV and the subscales of the DASS21 questionnaire in the euglycemic and prediabetic participants separately. To our knowledge, this is the first study to examine depression, anxiety, and stress levels in relation to body composition and HRV in prediabetic vs. euglycemic adolescents.

Numerous studies have found that prediabetes is strongly associated with increased weight and BMI [24,25]. Other studies report emotional stress and emotional eating, leading to disruptive behaviors that are linked to increased weight, BMI [26,27], abnormal metabolic profiles, and inflammatory body composition phenotypes [3,8,18]. Interestingly, such conclusions cannot be drawn from the demographic, psychometric, and body composition profile of our study sample. Although the prediabetic group, in relation with the euglycemic group, was overweight—though not to a statistically significant degree—body composition parameters did not differ significantly between the two groups.

Prediabetes is a stage where most of the macro- and micro-vascular lesions exhibit their insidious onset. One of the most dangerous and frequent complications is autonomic neuropathy. One facet of diabetic autonomic neuropathy is the cardiac autonomic neuropathy, as a result of profuse subclinical inflammatory processes that are also present in hyperglycemia [28]. Heart rate variability is a measure of sympathovagal imbalance, and it has been long used for the evaluation of the function of autonomic nervous system, either for research purposes or in the clinical setting [29].

One recent study demonstrated the effectiveness of HRV in conjunction with salivary cortisol in assessing stress levels. They claimed that the robustness of this method renders it a potential indication of future health [30]. Another study indicated that reduced HRV with higher psychological distress and increased salivary cortisol levels were observed in patients with temporomandibular disorders [31,32]. Another finding of the present study was the lack of significant differences between the two groups in the time and frequency domain parameters of the HRV, and this is in agreement with the results of Lee et al. in a study of a large number of obese prediabetic adolescents that are similar to ours [33]. Additionally, Asghar et al. demonstrated normal cardiac sympathetic innervation in prediabetic adults [34], a finding that reinforces our findings. The inflammatory processes, caused in the prediabetic state, may need to persist in time before producing clinically significant signs of cardiac autonomic neuropathy that may be detectable with an HRV test. Additionally, a surge of growth hormone is observed in adolescence. Growth hormone is responsible for the growth spurt but it also exhibits antioxidant properties [35] that possibly, even if it provokes insulin resistance [36], counterbalances the pro-oxidative processes of hyperglycemia [37]. Regrettably, no such studies exist in adolescents. However, heart rate variability is abnormal in adult patients with growth hormone deficiency. The impaired sympathetic tone could be a consequence of reduced central sympathetic tone or altered cardiac responsiveness to autonomic control and may contribute to the increased cardiovascular risk in those patients [38].

There seem to be biological sex differences in the hypothalamic-pituitary-adrenal (HPA) axis response in the normal physiology of the stress autonomic response, and thus in HRV results [39], due to gonadal steroids [40]. The release of cortisol differs between the sexes, particularly following stress events. Cortisol increases are higher and remain elevated for longer in females [41]. Both estrogen and testosterone temper with cortisol concentrations, and thus, the degree of HPA axis activation is also dependable on both of these hormones, suggesting an interaction between the HPA and the hypothalamic-pituitary-gonadal (HPG) axes. In addition to explaining the slightly increased, basal and stress-stimulated HPA axis function in the female sex, the estrogen-induced enhancement of the cortisol releasing hormone (CRH) neuron may also help explain the paradox of negative estrogen feedback effect on the hypothalamic gonadotropin-releasing hormone (GnRH) neuron, which, unlike the paraventricular nucleus, lacks estrogen receptors [42]. Conclusively, gonadal steroids impact HPA axis reactivity differentially. Gonadectomy of male rats elevates, while androgen replacement blunts the cortisol and adrenocorticotrophic hormone (ACTH) response to stress [43]. In contrast, ovariectomy reduces, while estradiol treatment increases, the gain of the HPA axis [44]. Interestingly, one study demonstrated that females when stressed presented with variants that did not differ on aggression, and secondary variants showed higher cortisol, testosterone, cortisol-to-dehydroepiandrosterone (DHEA) ratios, and HRV. These findings suggested that the neurobiological mechanisms underpinning aggression and thus, stress response may differ between women on primary versus secondary developmental pathways [31].

Our results suggested sexual dimorphism in the HRV tests. Females seemed to have an upregulated HPA axis, probably due to the rise of sex hormones, such as estrogens and progesterone [45]. Nevertheless, they did not exhibit statistically significant differences in any other study variables, such as the DASS21 subscales. In line with our results are the findings of the meta-analysis of Koenig and Thayer on biological sex differences in HRV [46]. These authors found that females showed less variability within the time-series of heartbeats indexed by SDNN and lower total power in the spectral density, probably due to a greater mean heart rate (HR) reflected by a smaller mean R-to-R (RR) interval.

Moreover, profound anatomic brain reorganization processes also take place in adolescence; part of these processes is known as “neural pruning”. The maturation of the reproductive system is accompanied by rising concentrations of the gonadal steroids. The human brain has a high density of steroid receptors, and the sex steroids exert various effects on neural networks during adolescence [47,48]. According to this model, sex steroids affect the development of the adolescent brain by sensitizing neural networks that result in a permanent reorganization of the brain. Sex steroids have different effects on the developing HPA axis in males and females: the rise of androgens in males inhibits the hypothalamic secretion of corticotropin-releasing hormone (CRH), while estrogens in females upregulate the HPA axis. Therefore, estrogens render females more susceptible to stress and stress disorders, while androgens make males more resilient to it [49], due to the estrogen response element in the glucocorticoid receptors [50]. Another aspect that should be considered is the developing heart of the adolescents and the existence of heart volume between males and females [51].

On a different note, interesting correlations between the HRV parameters and the psychometric subscales of DASS21 were revealed. HRV has been subject to criticism [52], since there are no clear boundaries between the two branches of the autonomic nervous system, due to their dynamic balance, especially in developing brains [53]. Normal, healthy adolescent brains give rise to odd results in the HRV tests. A significant number of studies that evaluated HRV parameters in adolescents concluded that these unusual findings may be attributed to changes in the prefrontal cortex and limbic system connections that transpire across adolescence [54].

Given the coupling of central and autonomic nervous system functioning in the regulation of the stress response, the decrease in vagotonic activity across adolescence may be driven, at least partially, by the central nervous system, as well as by the maturation of the sympathetic nervous system [55]. The DASS21 scores of anxiety in the euglycemic group seemed to decrease when the activity of the sympathetic nervous system increased. The results of the frequency domain of the HRV in the euglycemic group reflect the aforementioned findings. On the contrary, in the prediabetic group, the results were similar to those of adults. More specifically, when the function of the sympathetic nervous system was augmented, or when the sympathovagal balance was dysregulated, the stress, anxiety, and depression levels were also augmented. Whether this is due to maturity of the prefrontal cortex or IFG remains to be elucidated. Possibly, a combined approach of the activity of the LF and HF bands in HRV tests would result in more accurate interpretation of mental and physical stress, especially in periods of increased risk for the onset of emotional disorders, such as adolescence [56,57,58], and this is one limitation of this study. The small number of subjects in this study represents another limitation in this study.

## 5. Conclusions

Conclusively, impaired fasting glycemia seems to have no impact on body composition, mental health, or HRV; the participants of both groups had similar mental health scores, evaluated by the DASS21 questionnaire; body composition, evaluated by the BIA-ACC device; and HRV parameters, evaluated by PPG-Stress Flow. Adolescent females seem to have an upregulated HPA axis, as the values of HRV parameters were abnormal when compared with their male counterparts, independently of hyperglycemia. Thus, females seem to be more prone to stress-related disorders from a young age, but there are still a lot of missing parameters to be further evaluated to have definite conclusions. Any firm conclusions cannot be drawn from this study. Future, more sophisticated studies of larger numbers of prediabetic adolescents are needed to confirm these findings.

## Figures and Tables

**Table 1:** Characteristics of the study sample.

Variables	Euglycemic Group IQR (*n* = 16)	Prediabetic Group IQR (*n* = 13)	*p*
Age (years)	14.5 (12.5–15.75)	15 (15–16.5)	0.17
Male sex	6 (37.5%)	7 (53.8%)	0.379
Weight Z-score	0.68 (0.05–2.17)	2.07 (−0.03–2.3)	0.2
Height Z-score	0 (−0.14–0.93)	0.6 (0.05–1.75)	0.13
BMI Z-score	0.7 (−0.1–1.9)	1.76 (0.05–2.15)	0.3
1st visit FBG (mg/dL)	92 (83.5–97)	105 (102–111)	0.003
2nd visit FBG (mg/dL)	93 (87.2–96.7)	110 (105–118)	<0.001
3rd visit FBG (mg/dL)	91 (80.5–99)	104 (101.5–108)	0.001
HbA1c (%)	5 (4.85–5.3)	5.2 (5–5.2)	0.252
Positive family history of diabetes	10 (62.5%)	10 (76.9%)	0.54
Single-parent family	3 (18.8%)	3 (32.1%)	0.775
DASS 21 Stress Subscale	13 (3–23.5)	15 (6.5–19.5)	0.77
DASS 21 Anxiety Subscale	12 (0.5–19)	8 (2.5–14)	0.6
DASS 21 Depression Subscale	10 (1–20)	10 (2.5–15.5)	0.86

Abbreviations/Clarifications: FBG: fasting blood glucose; positive family history of diabetes: presence of a first or second-degree relative with diabetes; and single-parent family: divorced, separated, or widowed legal guardians. Results are presented as median (interquartile range).

**Table 2:** Body composition and heart rate variability indices of the two study groups.

Variables	Euglycemic Group IQR (*n* = 16)	Prediabetic Group IQR (*n* = 13)	Statistical Significance
Total Body Water (Body Weight %)	50 (46–58)	49 (43.5–53)	0.455
Total Body Water (L)	28.5 (26–36)	40.6 (27.25–45.3)	0.144
Extracellular Water (Body Weight %)	45 (42–47.5)	40 (39–46)	0.14
Extracellular Water (L)	13.1 (12.3–15.7)	15.9 (12.75–17.5)	0.124
Intracellular Water (Body Weight %)	55 (52.5–58)	60 (54–61)	0.14
Intracellular Water (L)	15.6 (14.1–20.9)	24.3 (14.75–27.75)	0.124
Fat-Free Mass (Body Weight %)	72 (68.5–87.5)	68 (59.5–81)	0.174
Fat-Free Mass (kg)	44.1 (41.15–52.7)	55.5 (43.9–61.85)	0.112
Fat Mass (Body Weight %)	28 (12.5–32)	32 (19–40.5)	0.174
Fat Mass (kg)	15.6 (6.55–25.1)	28.9 (10.5–38.55)	0.112
Skeletal Muscle Mass (kg)	14.6 (13.1–20.05)	23.4 (13.75–26.95)	0.124
Skeletal Muscle Mass (Fat-Free Mass %)	33.1 (31.45–40.15)	40.3 (34.55–44.75)	0.151
Abdominal Adipose Tissue (cm^2^)	231.65 (115.075–437.65)	470.25 (119.7–650.48)	0.204
Intramuscular Adipose Tissue (kg)	0.8 (0.6–1.5)	1.75 (0.75–2.2)	0.108
Intramuscular Adipose Tissue (Body Weight %)	1.5 (1–1.8)	1.95 (1.15–2.35)	0.116
Mean Heart Rate	76.1 (69.33–78.58)	77.3 (74.5–89.5)	0.172
SDNN (ms)	106 (75.75–201.5)	82 (52–117.5)	0.238
RMSSD (ms)	104 (56–267.25)	104 (39–115.5)	0.306
Scatter area (ms^2^)	30,852.5 (11,185.75–32,269.5)	20,778 (5702.5–36,513)	0.239
TOTAL POWER (ms^2^/Hz)	9.275 (8.5–9.95)	8.7 (7.89–9.195)	0.154
VLF POWER (ms^2^/Hz)	8.05 (7.46–8.66)	7.12 (6.87–7.87)	0.107
LF POWER (ms^2^/Hz)	7.97 (7.49–8.74)	7.14 (6.9–8.51)	0.193
HF POWER (ms^2^/Hz)	8.335 (7.04–9.2)	7.82 (6.51–8.275)	0.154
LF/HF Log	−0.4 (–0.625–0.125)	−0.1 (–0.7–0.55)	0.619
LF/HF Ratio	0.7 (0.575–1.25)	0.9 (0.5–1.7)	0.732
LF %	40.4 (35–52.13)	47.9 (32.7–63)	0.762
HF %	59.6 (47.88–65)	52.1 (37–67.3)	0.762

Abbreviations: SDNN: standard deviation of normal-to-normal intervals; RMSSD: root mean square of successive differences; VLF: very low frequency; LF: low frequency; HF: high frequency; and ms: milliseconds. Results are presented as median (interquartile range).

## Abbreviations

ADA	American Diabetes Association
BIA	Bio-impedance analysis
BMI	Body mass index
CAM	Center for Adolescent Medicine
CRH	Corticotropin-releasing hormone
DASS21	Depression, anxiety, stress scales 21
DXA	Dual-energy X-ray absorptiometry
HRV	Heart rate variability
Hb1Ac	Hemoglobin A1c
IFG	Impaired fasting glycemia
IGT	Impaired glucose tolerance
HR	Heart rate
HF	High frequency
HPA	Hypothalamic-pituitary-adrenal axis
LF	Low frequency
PFC	Prefrontal Cortex
SAM	Sympatho-adrenal medullary system
T1DM	Type 1 diabetes
T2DM	Type 2 diabetes
RMSSD	Square root of the mean of the sum of the squares of differences between adjacent Normal to Normal intervals
SDNN	Standard deviation of all Normal to Normal intervals
VLF	Very low frequency
